# Enhanced HSC-like cell generation from mouse pluripotent stem cells in a 3D induction system cocultured with stromal cells

**DOI:** 10.1186/s13287-021-02434-2

**Published:** 2021-06-19

**Authors:** Wei Shan, Qin Yu, Yan Long, Qian Luo, Honghu Li, Yingli Han, Yulin Xu, Shan Fu, Xiangjun Zeng, Cong Wei, Yang Gao, Xiaoqing Li, Xia Li, Lifei Zhang, Lizhen Liu, Ming Chen, Pengxu Qian, He Huang

**Affiliations:** 1 Bone Marrow Transplantation Center, The First Affiliated Hospital, School of Medicine, Zhejiang University, No.79 Qingchun Road, Hangzhou, Zhejiang PR China; 2 Institute of Hematology, Zhejiang University, Hangzhou, Zhejiang PR China; 3 Zhejiang Engineering Laboratory for Stem Cell and Immunotherapy, Hangzhou, Zhejiang PR China; 4 Zhejiang Laboratory for Systems & Precison Medicine, Zhejiang University Medical Center, Hangzhou, Zhejiang PR China; 5 College of Life Science, Zhejiang Chinese Medical University, Hangzhou, Zhejiang 310053 PR China; 6 Department of Hematology, Sir Run Run Shaw Hospital, Zhejiang University School of Medicine, No. 3 Qingchun East Rd., Hangzhou, 310016 Zhejiang PR China; 7 Department of Bioinformatics, College of Life Sciences, Zhejiang University, Hangzhou, 310058 China; 8 Center of Stem Cell and Regenerative Medicine, School of Medicine, Zhejiang University, Hangzhou, 310012 PR China

**Keywords:** 3D system, Pluripotent stem cells, Hematopoiesis, CD201, Notch

## Abstract

**Background:**

Decades of efforts have attempted to differentiate the pluripotent stem cells (PSCs) into truly functional hematopoietic stem cells (HSCs), yet the problems of low differentiation efficiency in vitro and poor hematopoiesis reconstitution in vivo still exist, mainly attributing to the lack of solid, reproduced, or pursued differentiation system.

**Methods:**

In this study, we established an in vitro differentiation system yielding in vivo hematopoietic reconstitution hematopoietic cells from mouse PSCs through a 3D induction system followed by coculture with OP9 stromal cells. The in vivo hematopoietic reconstitution potential of c-kit^+^ cells derived from the mouse PSCs was evaluated via m-NSG transplantation assay. Flow cytometry analysis, RNA-seq, and cell cycle analysis were used to detect the in vitro hematopoietic ability of endothelial protein C receptor (EPCR, CD201) cells generated in our induction system.

**Results:**

The c-kit^+^ cells from 3D self-assembling peptide induction system followed by the OP9 coculture system possessed apparently superiority in terms of in vivo repopulating activity than that of 3D induction system followed by the 0.1% gelatin culture. We interestingly found that our 3D+OP9 system enriched a higher percentage of CD201^+^c-kit^+^cells that showed more similar HSC-like features such as transcriptome level and CFU formation ability than CD201^-^c-kit^+^cells, which have not been reported in the field of mouse PSCs hematopoietic differentiation. Moreover, CD201^+^ hematopoietic cells remained in a relatively slow cycling state, consistent with high expression levels of P57 and Ccng2. Further, we innovatively demonstrated that notch signaling pathway is responsible for in vitro CD201^+^ hematopoietic cell induction from mouse PSCs.

**Conclusions:**

Altogether, our findings lay a foundation for improving the efficiency of hematopoietic differentiation and generating in vivo functional HSC-like cells from mouse PSCs for clinical application.

**Supplementary Information:**

The online version contains supplementary material available at 10.1186/s13287-021-02434-2.

## Background

Currently, allogeneic HSC transplantation has been widely used in a clinical setting, yet allogeneic transplantation often leads to graft versus host disease (GVHD) [1]. Although hematopoietic stem and progenitor cells (HSPCs) enable autologous treatment of blood disorders, de novo generation of hematopoietic cells from pluripotent stem cells still compromises the unlimited high yield and rich hematopoiesis engraftment potential [2]. To generate functional hematopoietic stem cells (HSCs) from pluripotent stem cells (PSCs) in vitro, many attempts involving transcription factor (TF)- or embryonic bodies (EBs)-mediated differentiation, stromal cell coculture, and teratoma-based methods have been made [3, 4], yet a viable and reproducible approach is still lacking, and previous protocols need further refinement. Although two recent studies had revealed that the inducible HSC (iHSC) derived from PSCs and adult endothelium through over-expression of hematopoietic transcription factor possessed long-term reconstitution (LTR) potential in mouse hosts [2, 5], the enforced expression of factors via lenti- or retro-viral transduction approaches conferred the tumorigenic risk to the iHSC [6].

OP9 stromal cells have been reported to augment the survival of hematopoietic precursors and progenitors derived from human embryonic stem cells (ESCs) [7]; moreover, they also promote the hematopoietic differentiation of mouse ESCs and favor the development of definitive HSCs from pre-HSCs in the mouse aorta-gonad-mesonephros (AGM) region [8]. In fact, only using the OP9 co-culture system combined with hematopoietic related cytokines, we hardly obtained the in vivo functional reconstitution hematopoietic cells from PSCs [9]. In this study, we innovatively used the OP9 coculture system to establish a 3D self-assembling peptide-mediated OP9 coculture system that gives rise to a high ratio of induced hematopoietic cells (iHCs) with in vivo hematopoiesis reconstitution in both peripheral blood (PB) and bone marrow (BM) in immunodeficient mice, which provides infinite functional iHCs from PSCs without involving the tumorigenic risk caused by virus transduction approaches and an invaluable platform to study mouse hematopoiesis development in vitro.

Recent studies claimed that the endothelial protein C receptor (EPCR, CD201) gene, a novel marker, can enrich mouse HSCs within AGM regions and the fetal liver and can even enrich human HSCs within cord blood and the fetal liver [10–13]. In our optimized induction system, we first identified that the CD201^+^ cluster of hematopoietic cells was highly prevalent and showed similar characteristics to the mouse embryonic and adult CD201^+^ HSC with LTR capacity. Interestingly, the CD201^+^ cell populations derived from mouse PSCs are superior in terms of hematopoietic TF, markers, colony-forming unit (CFU) colonies, and hematopoietic related regulation signaling pathways. More importantly, we innovatively demonstrated that notch signaling pathway participated in the CD201^+^ hematopoietic cell generation from mouse PSCs. In sum, for the first time, we established a novel approach for generating functional hematopoietic cells, which technically creates a link between the unlimited PSCs source and HSC-based immunotherapy for translational purposes.

## Methods

### The mESC culture

Mouse ESCs derived from 129/ola and C57BL/6 mice (Shanghai Institute of Biochemistry and Cell Biology) were maintained on inactivated mouse embryonic fibroblasts (MEFs) in mESC medium containing DMEM/F12 (Invitrogen) supplemented with 15% fetal bovine serum (FBS, Gibco), 1% nonessential amino acids (Invitrogen), 1% GlutaMAX (Invitrogen), 0.1 mM 2-mercaptoethanol (Invitrogen), 1000 U/mL leukemia inhibitor factor (Biolead), and penicillin/streptomycin (Solarbio® LIFE SCIENCES). OP9 and OP9-DL1 stromal cells (gifts from Professor Jinyong Wang, State Key Lab of GuangZhou Institutes of Biomedicine and Health, Chinese Academy of Sciences) were cultured with α-MEM (Gibco) supplemented with 20% FBS (Gibco).

### Hematopoietic differentiation of mouse PSCs using a 3D self-assembling peptide induction system followed by an OP9 coculture system

For stage I, the 3D induction protocol involved EBs hematopoietic differentiation as previously described [3]. For stage II, 1 × 10^5^ OP9 stromal cells were plated in 0.1% gelatin-coated 12-well plate and were treated with mitomycin C after 24-h culture. Then, 20 6–8-day hematopoietic-like colonies derived from EBs in the 3D induction system were picked and reseeded on OP9 stromal cells for further hematopoietic differentiation. The medium for the OP9 coculture system was composed of a-MEM (Gibco), 10% FBS (Gibco), and cytokines (100 ng/mL stem cell factor (SCF), 100 ng/mL IL-3, and 100 ng/mL Flt3 ligand, all from PeproTech) [14].

For experiments involving dual antiplatelet therapy (DAPT, Notch inhibition), DAPT was added to day 0 OP9 coculture system medium (20μm; Selleck) [15], while corresponding control conditions had DMSO added at 1:500. After 5 days’ coculture, cells were collected for flow cytometry analysis.

### Flow cytometry analysis and cell sorting

Cells derived from in vitro mouse PSC hematopoietic differentiation and the in vivo PB and BM of m-NSG mice were harvested and suspended in PBS with 2% FBS. Before antibody incubation, the cells were blocked with an anti-CD16/32 antibody (eBioscience). The following antibodies were used for analysis: Alexa Fluor 700 (AF700)-conjugated anti-Flk1 (eBioscience), allophycocyanin (APC)-conjugated anti-TIE2 (eBioscience), APC-Cy7-conjugated anti-c-kit (eBioscience), AF700-conjugated anti-Lineage (eBioscience), APC-conjugated anti-Sca-1 (eBioscience), peridinin-chlorophyll protein (PerCP)-eFluor® 710-conjugated anti-CD201 (eBioscience), APC-Cy7-conjugated anti-TER119 (eBioscience), PerCP-Cy5.5-conjugated anti-CD45.1 (eBioscience), and PE-Cy7-conjugated anti-CD45.2 (eBioscience). The following antibodies were used for sorting: APC-Cy7-conjugated anti-c-kit (eBioscience), eFluor450-conjugated anti-Lineage (eBioscience), and phycoerythrin (PE)-conjugated anti-CD201 (eBioscience). Samples were measured by BD Fortessa, and cells were sorted by BD Aria II. The data were analyzed using FlowJo Version 10 software.

### Colony-forming unit (CFU) assay and May-Giemsa staining

CFU assays were performed by plating 2 × 10^4^ cells into MethoCult™ GF M3434 medium (Stem Cell Technologies, Inc.) in a 35-mm culture dish for 12 days. The colonies were counted based on standard morphological criteria. BFU-E (burst-forming unit-erythroid), CFU-GM (colony-forming unit-granulocyte/macrophage), and CFU-GEMM (colony-forming unit-granulocyte/erythroid/macrophage/megakaryocyte) were classified and enumerated based on morphological recognition. In addition, colonies were picked, fixed on glass slides, and stained with Giemsa solution (Sigma).

### Cell cycle analysis

Cells were harvested and suspended in PBS with 2% FBS. Then, the cells were stained with HSC-related antibodies as described above. Cells were fixed and permeabilized with eBioscience^TM^ Fixation/Permeabilization Concentrate (Invitrogen) at room temperature (RT) for 20 min in the dark. After washing with PBS containing 2% FBS, cells were incubated with PE-Cy7-conjugated anti-Ki-67 antibody (eBioscience) at RT for 30 min in the dark. Later, washed cells were incubated with 4′,6-diamidino-2-phenylindole (DAPI) (Biogems) at RT for 30–40 min, followed by flow cytometry analysis with BD Fortessa. The data were analyzed using FlowJo Version 10 software.

### Mouse transplantation assay

Female m-NSG mice aged at 6–8 weeks were purchased from Shanghai Model Organisms Center, Inc., maintained in the standard SPF animal house and used in all studies [16]. Mouse PSC-derived sorted c-kit^+^ cells were intrafemorally injected into each irradiated (2.25 Gy) m-NSG mouse. The mice were fed water containing Baytril (Bayer) for 2 weeks to prevent infection. All animal work was supported by The Institutional Animal Care and Use Committee of Zhejiang University.

### Quantitative reverse transcription polymerase chain reaction

Quantitative polymerase chain reaction (qPCR) was performed as follows: day 5 mouse PSC-derived total cells in 3D+OP9 coculture system in the presence of DMSO or DAPT were collected. Total RNA was extracted from using Trizol reagent (Invitrogen) and 1 μg RNA was reverse-transcribed into complementary DNA (cDNA) using PrimeScript RT reagent Kit (Takara) according to the manufacturer’s instructions. qPCR was completed in a Light Cycler system (Roche) Q5 using SYBR Premix Ex Taq (Takara). Each sample was performed in triplicate and all results were normalized to the expression of Actin. Fold expression relative to the reference gene was calculated using the comparative method 2^−ΔΔCt^. PCR primers used for reverse transcription PCR are referenced to Table 1.

**Table 1:** Sequences of the primers used for quantitative PCR

Gene	Sequence, 5′-3′
Hey1	
Forward	CCGACGAGACCGAATCAATAAC
Reverse	TCAGGTGATCCACAGTCATCTG
DLL1	
Forward	GCAGGACCTTCTTTCGCGTAT
Reverse	AAGGGGAATCGGATGGGGTT
DLL4	
Forward	TTCCAGGCAACCTTCTCCGA
Reverse	ACTGCCGCTATTCTTGTCCC
Notch1	
Forward	GATGGCCTCAATGGGTACAAG
Reverse	TCGTTGTTGTTGATGTCACAGT
Notch4	
Forward	GAACGCGACATCAACGAGTG
Reverse	GGAACCCAAGGTGTTATGGCA
Actin	
Forward	ACGTAGCCATCCAGGCTGGTG
Reverse	TGGCGTGAGGGAGAGCAT

### RNA-seq

Total RNA was extracted from sorted cell samples using TRIzol Reagent (Life Technologies) following the manufacturer’s instructions. cDNA libraries were constructed using the VAHTS mRNA-seq v2 Library Prep Kit for Illumina (Vazyme) according to the manufacturer’s protocol. Library sequencing was performed on an Illumina HiSeq X Ten platform (Illumina) to generate 150-bp paired-end reads. The paired-end reads were processed and aligned to the reference genome (GRCm38) using HISAT2 (v.2.0.5). Mapped reads for each sample were assembled into transcriptome data using StringTie (v.1.3.3) with a reference-based approach. The normalized expression value was represented by fragments per kilobase of transcript per million mapped reads (FPKMs). Cuffdiff (v1.3.0) was used to calculate the differential expression genes for each sample. Only comparisons with q-values < 0.05 and absolute log2 (fold change) values ≥1 were considered as significantly differentially expressed genes. Functional enrichment analysis of DEGs was performed with DAVID (https://david.ncifcrf.gov/) and -log10(*p* value) were plotted to show term significance.

The RNA-seq data are available at Gene Expression Omnibus (GEO) (accession number: GSE175563).

### Statistical analysis

The number of biological replicates is indicated by the n value. All graphs depict mean ± SD. Statistical analysis was performed using a two-tailed un-paired Student’s test. The results were considered statistically significant at *P* value < 0.05 and were denoted as NS, not significant; * *P* < 0.05; ** *P* < 0.01; *** *P* < 0.001. The statistical analysis data were assessed using GraphPad Prism 8.

## Results

### The steady promoting hematopoietic differentiation system was established using a 3D self-assembling peptide induction system followed by an OP9 coculture system

**Fig. 1:**
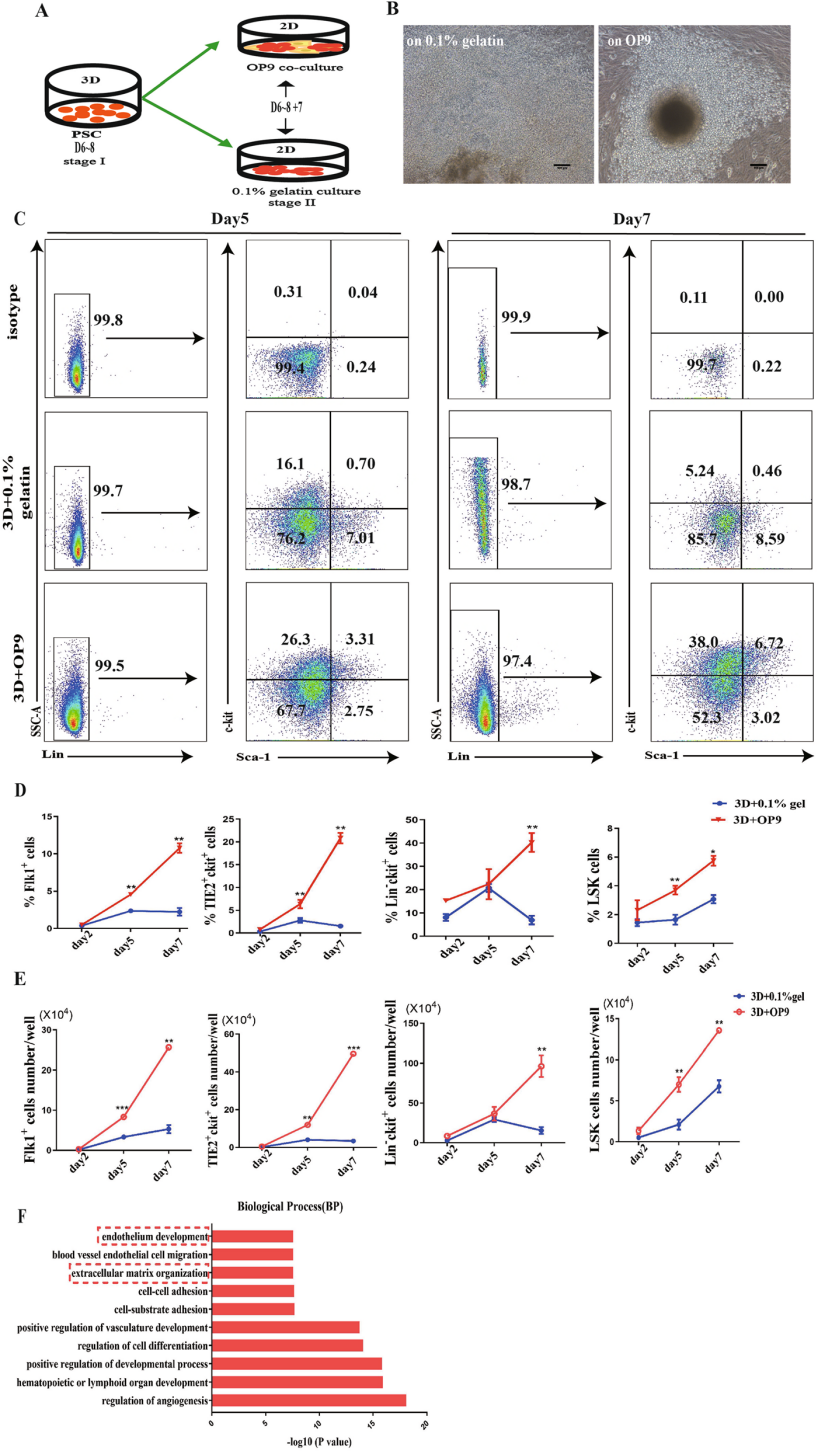
The 3D self-assembling peptide induction system followed by the OP9 coculture system promotes hematopoietic differentiation of mouse PSCs. **A** Schematic overview of mouse PSCs hematopoietic differentiation in the 3D self-assembling peptide induction system followed by the OP9 coculture system. **B** Representative morphology of hematopoietic cells from hematopoietic-like colonies derived from EBs in a 3D induction system followed by coculture with OP9 stromal cells and 0.1% gelatin. **C** Representative flow cytometry analysis of the percentage of Lin^−^Sca-1^+^c-kit^+^(LSK) on day 5 and day 7 respectively (*n* = 3). **D** Statistical analysis of percentage of mesoderm cells (Flk1^+^), hemogenic endothelium (TIE2^+^c-kit^+^), Lin^−^c-kit^+^ and LSK on day 2, day 5, and day 7 respectively (*n* = 3). **E** Absolute numbers of Flk1^+^, TIE2^+^c-kit^+^, Lin^−^c-kit^+^, and LSK cells in between 3D+OP9 and 3D+0.1% gelatin on day 2, day 5, and day 7 respectively (*n* = 3). **F** Representative statistically enriched pathways of the upregulated genes in 3D+OP9 group compared with 3D+0.1% gelatin group. Data are the means ± SD from three independent experiments. Error bars represent mean ± SD of samples from three independent experiments (*n* = 3). NS, not significant; *p < 0.05, **p < 0.01, ***p < 0.001

We previously utilized the 3D self-assembling peptide to establish the hematopoietic differentiation approach with a method that is difficult to operate, where 3D system-derived cells were wrapped by the hydrogel as well as the primary generation of hematopoietic precursor cells. To further optimize the differentiation method to establish a viable and reproducible hematopoietic differentiation system, we used the OP9 stromal cells described to support the maturation of hematopoietic precursor cells into definitive HSCs to obtain more efficient hematopoietic differentiation and more HSCs and HSPCs. In our study, we first investigated the hematopoietic differentiation effect of a 3D self-assembling peptide induction system followed by an OP9 coculture system. After 6–8 days of EB-mediated 3D hematopoietic induction, OP9 stromal cells were cocultured with 3D system-derived hematopoietic-like colonies (Fig. 1A). A previous study reported that the OP9 coculture system composed of SCF, IL3, and the Flt3 ligand promoted the development of hematopoietic precursor cells into HSCs. Likewise, in our differentiation system, on day 5 after coculture with hematopoietic-like colonies, we found that cobblestone-like cells were more apparent in the OP9 coculture system than in the differentiation system of hematopoietic-like colonies cultured on 0.1% gelatin (3D+OP9 or 3D+0.1% gelatin) (Fig. 1B). Flk1^+^ mesoderm cells and TIE2^+^c-kit^+^ hemogenic endothelial cells maintained an increasing tendency from day 2 to day 7 in our induction system. Flow cytometry analysis data suggested that the 3D+OP9 coculture obtained a higher percentage of Flk1^+^ and TIE2^+^c-kit^+^ cells than 3D+0.1% gelatin coculture (Fig. 1D). Furthermore, Lin^−^c-kit^+^ cells and Lin^−^Sca-1^+^c-kit^+^ cells marking the HSC/HSPC cluster obtained a greater rate in the group of 3D+OP9 coculture than 3D+0.1% gelatin coculture (Fig. 1C–E). Then, RNA-seq results showed that the biological functions of genes upregulated in day 5 total cells from 3D+OP9 coculture as compared to 3D+0.1% gelatin coculture were more related with hematopoietic organ development and extracellular matrix organization (Fig. 1F). These results suggest that OP9 stromal cells play a critical role in optimizing the previously established 3D self-assembling peptide-mediated induction system, and we established a viable and repeatable 3D self-assembling peptide-mediated OP9 coculture system.

### Comparison between the in vivo repopulating activity of hematopoietic cells from the 3D self-assembling peptide induction system followed by the OP9 coculture system and 0.1% gelatin

**Fig. 2:**
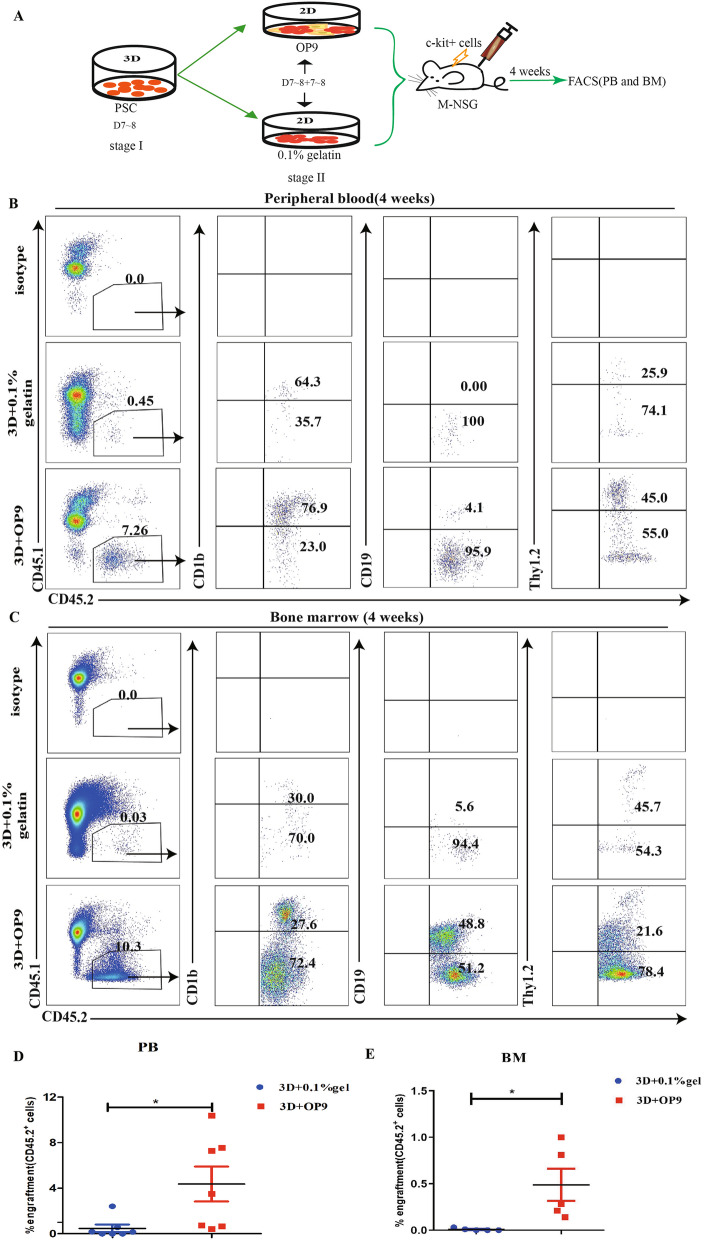
In vivo transplanted potential of 3D+OP9 and 3D+0.1% gelatin induction system-derived c-kit^+^ hematopoietic cells. **A** Schematic representation of the transplantation strategy. **B**–**E** Representative flow cytometric plots for CD45.1 and CD45.2 expression in the PB and BM from m-NSG recipient mice (CD45.1), meanwhile representative flow cytometric plots for expression of CD11b, CD19, and thy1.2 in gated CD45.2^+^ cells. **F**–**G** Percentage CD45.2^+^ chimerism in the PB (left) and BM (right) from m-NSG. Data are the means ± SD from three independent experiments. Error bars represent mean ± SD of samples from at least three independent experiments (n>=3). NS, not significant; *p < 0.05

Subsequently, we evaluated the hematopoiesis reconstitution potential of mouse PSC-derived hematopoietic cells from 3D+OP9 coculture system and 3D+0.1% gelatin coculture system. First, the frequency of Lin^−^c-kit^+^ cells induced by 3D+OP9 coculture system was slightly higher than that induced group by 3D+0.1% gelatin (Fig. 1C, D). Next, the in vivo reconstitution (STR) activity of inducible c-kit^+^ hematopoietic cells was compared. After transplantation of day 5 c-kit^+^ cells from 3D+OP9 and 3D+0.1% gelatin coculture system, CD45.2^+^ cells were detected for 4 weeks (Fig. 2A). m-NSG mice were sacrificed, and FACS analysis of the PB and BM of recipient mice showed that the c-kit^+^ cells from the 3D+OP9 coculture system showed higher levels of in vivo hematopoiesis than those from the 3D+0.1% gelatin group, with chimerism frequencies of 7.74% and 0.55% in the PB and 0.34% and 0.01% in the BM, respectively (Fig. 2B–E). We also examined the hematopoietic multilineages in sacrificed mice, including myeloid (CD45.2^+^CD11b^+^) and lymphoid (CD45.2^+^CD19^+^, CD45.2^+^Thy1.2^+^) hematopoietic lineages in surviving mice. The phenotypic analysis indicates that c-kit^+^ hematopoietic progenitors from the 3D+OP9 and 3D+0.1% gelatin groups all reconstituted myeloid and B lymphoid lineages and T cells, with the c-kit^+^ hematopoietic progenitors from 3D+OP9 inducible system possessing the stronger hematopoiesis superiority (Fig. 2F, G). Together, our results provide direct evidence that hematopoietic cells derived from the 3D+OP9-induced hematopoiesis system could reconstitute multilineage hematopoiesis in vivo, resembling natural hematopoietic cell development.

### CD201 enriches a cluster of functional Lineage-negative (Lin^−^) Sca-1^+^c-kit^+^ (LSK) HSCs/HSPCs in our optimized 3D self-assembling peptide-mediated OP9 coculture hematopoietic induction system

**Fig. 3:**
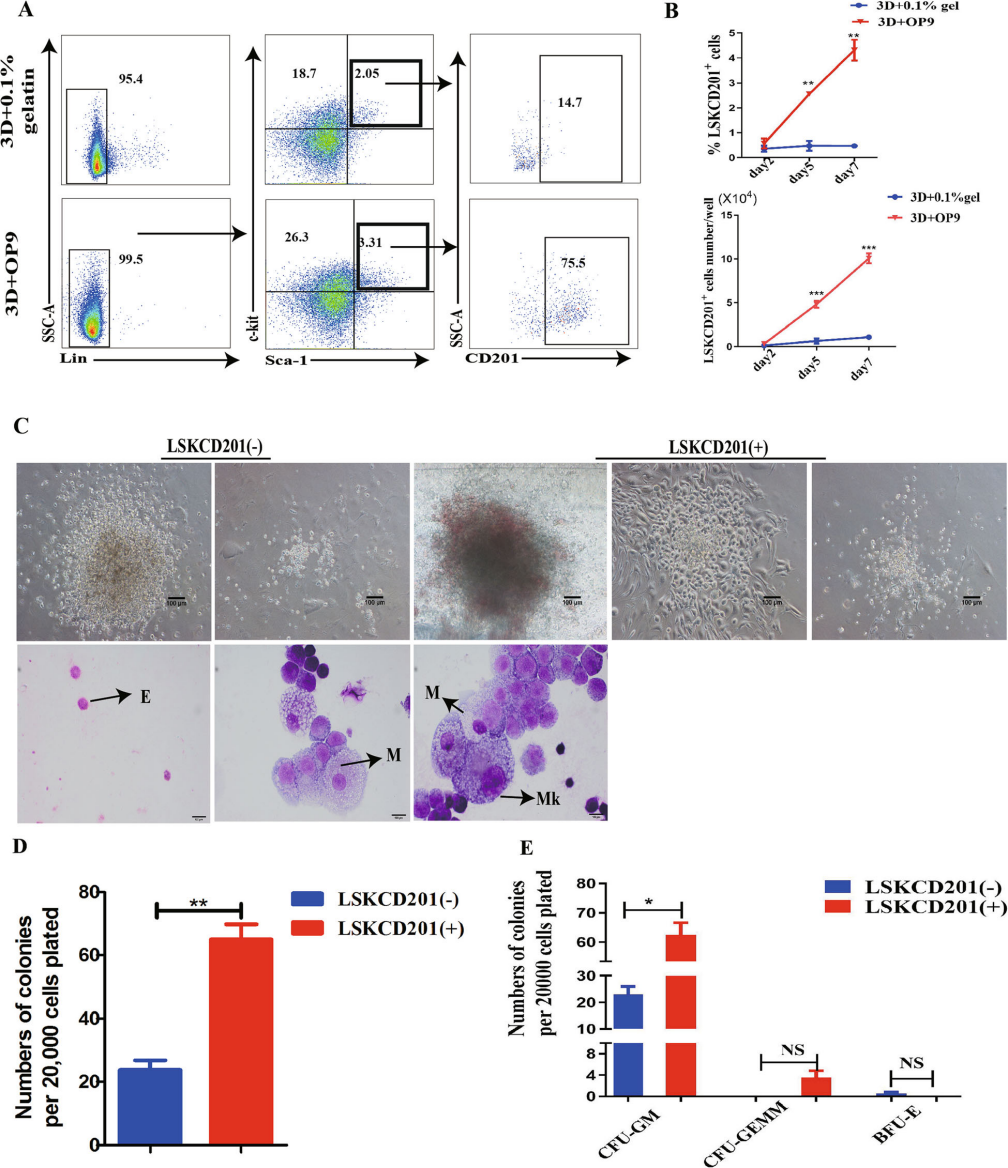
CD201 expression marks a cluster of functional HSC/HSPC in our 3D self-assembling peptide-mediated OP9 coculture hematopoietic induction system. **A** FACS analysis of the percentage of LSK and LSKCD201^+^ cells derived from 3D+OP9 and 3D+0.1% gelatin group on day 5. **B** Statistical analysis of percentage and absolute numbers LSKCD201^+^ cells of the 3D+OP9 and 3D+0.1% gelatin co-culture system on days 2, 5, and 7 respectively (*n* = 3). **C** Sorted Lin^−^c-kit^+^CD201^+^ cells were analyzed by a CFU assay. Representative field images of the different types of colonies obtained from Lin^−^c-kit^+^CD201^-^ and Lin^−^c-kit^+^CD201^+^ cells and cellular morphology of colonies by May-Giemsa staining. Arrowheads depict indicated morphology. E, erythrocyte; M, macrophage; Mk, megakaryocyte. Scale bar =100μm. **D** Lin^−^c-kit^+^CD201^+^ cells formed a significantly higher number of colonies than Lin^−^c-kit^+^CD201^-^ cells. **E** Statistical analysis of the variety of colonies between Lin^−^c-kit^+^CD201^-^ cells and Lin^−^c-kit^+^CD201^+^ cells. Error bars represent mean ± SD of samples from at least three independent experiments (*n* = 3). NS, not significant; *p < 0.05, **p < 0.01

To assess the hematopoietic potential of CD201^+^ cells in our differentiation system. Flow cytometry analysis was used to detect the CD201^+^ level of expression. It was found that the percentage CD201^+^ cell population was increased and reached approximately 50% on day 5 in the 3D+OP9 coculture differentiation system (Figure S1A-B). Further flow cytometry analysis revealed that roughly 75.5% of LSK cells were CD201^+^ within the group of 3D+OP9 coculture system, yet only 14.7 % of CD201^+^ cells within the LSK cell cluster from the group of 3D+0.1% gelatin coculture system, which suggested that the 3D self-assembling peptide-mediated OP9 coculture system promoted the generation of LSKCD201^+^ cells (Fig. 3A). Statistical analysis data confirmed that the 3D+OP9 coculture group had a higher percentage of LSKCD201^+^ cells than the 3D+0.1% gelatin coculture group on day 5 and day 7 (Fig. 3B). A CFU assay and morphological analysis were further conducted to evaluate the lineage differentiation potential of CD201^+^ and CD201^−^ cells. Sorted differentiated day 5 Lin^−^c-kit^+^CD201^+^ cells formed a significantly higher number of colonies than Lin^−^c-kit^+^CD201^−^ cells (Fig. 3C, D). In addition, Lin^−^c-kit^+^CD201^+^ cells formed a higher number of CFU granulocyte (CFU-G)-, CFU macrophage (CFU-M)-, CFU granulocyte macrophage (CFU-GM)-, and CFU granulocyte erythrocyte monocyte macrophage (CFU-GEMM)-derived colonies, whereas Lin^−^c-kit^+^CD201^−^ cells resulted in few CFU-G, CFU-M, CFU-GM, and CFU-GEMM colonies (Fig. 3E). Together, these data indicate that CD201^+^ hematopoietic cells from mouse PSCs possess more advantages in terms of hematopoiesis potential.

### Transcriptome analysis of LSKCD201^+^ cells from mouse PSCs in our hematopoietic differentiation system

**Fig. 4:**
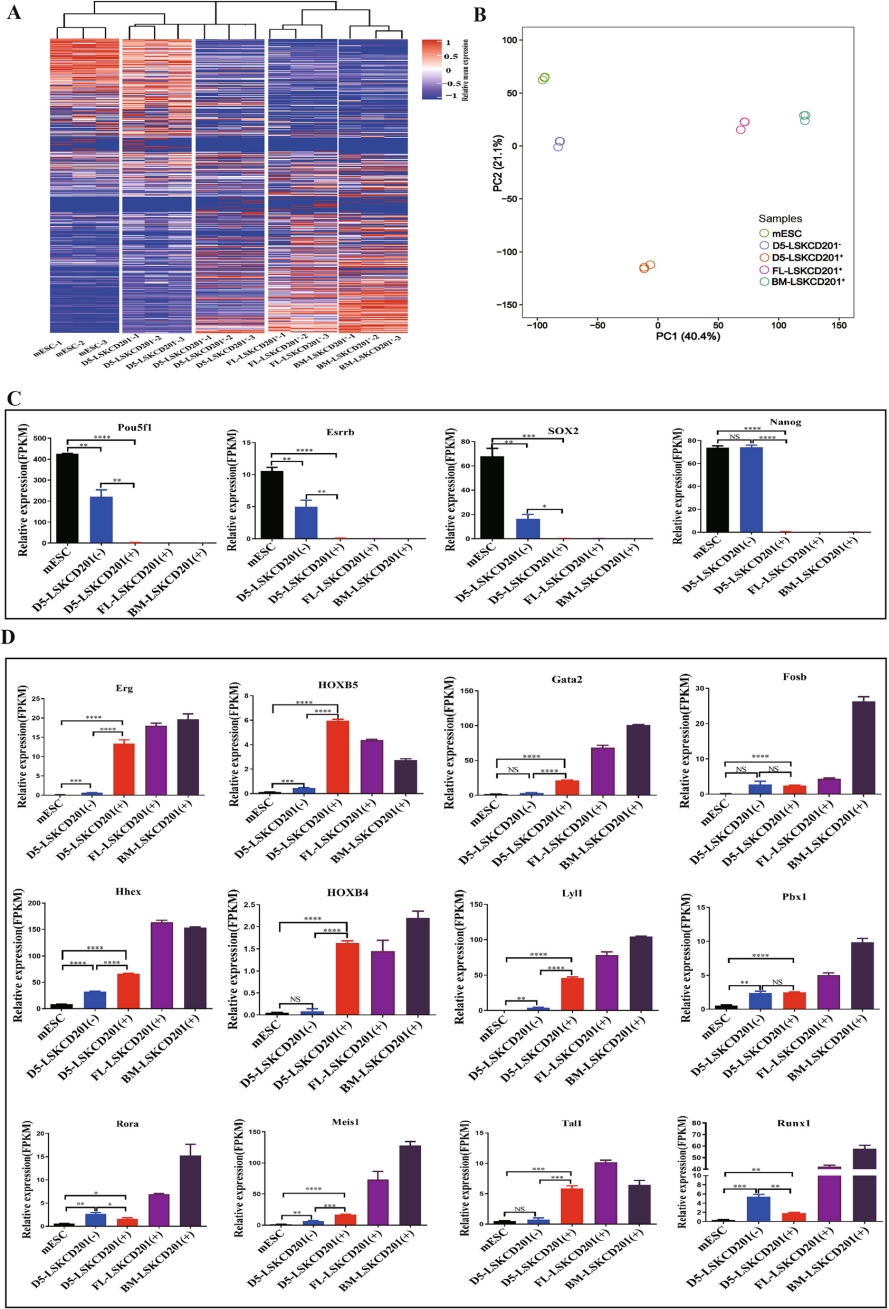
Global gene expression analysis of mouse PSCs, BM-LSKCD201^+^, FL-LSKCD201^+^ day 5 differentiated LSKCD201^+^ cells and LSKCD201^−^ cells (D5-LSKCD201^+^ and D5-LSKCD201^−^) of our hematopoietic induction system. **A** Hierarchical clustering of heatmaps of mouse PSCs, BM-LSKCD201^+^ cells, FL-LSKCD201^+^ cells, D5-LSKCD201^−^ cells, and D5-LSKCD201^+^ cells. **B** PCA plots of five cell groups of mouse PSCs, BM-LSKCD201^+^ cells, FL-LSKCD201^+^ cells, D5-LSKCD201^−^ cells, and D5-LSKCD201^+^ cells. **C** The expression of pluripotency-related genes in mouse PSCs, BM-LSKCD201^+^ cells, FL-LSKCD201^+^ cells, D5-LSKCD201^−^ cells, and D5-LSKCD201^+^ cells is shown as the FPKM values, Data are represented as mean ± SD (*n* = 3). **D** The expression of hematopoietic-related genes in mouse PSCs, BM-LSKCD201^+^ cells, FL-LSKCD201^+^ cells, D5-LSKCD201^-^ cells, and D5-LSKCD201^+^ cells is shown as the FPKM values. Data are represented as mean ± SD (*n* = 3)

We further analyzed the whole-genome transcriptomes of LSKCD201^+^ and LSKCD201^−^ on day 5 of our differentiation system. We performed RNA-seq analysis on mouse PSCs, day 5 coculture differentiated LSKCD201^+^ and LSKCD201^−^ cells (D5-LSKCD201^+^, D5-LSKCD201^−^), bone marrow-derived LSKCD201^+^ (BM-LSKCD201^+^) from C57BL/6 mice aged at 6–8 weeks, and fetal liver-derived LSKCD201^+^ (FL-LSKCD201^+^) cells from C57BL/6 mice on embryonic day 12.5 (E12.5). Compared with D5-LSKCD201^−^ cells, D5-LSKCD201^+^ cells showed a genome transcriptome more similar to that of BM-LSKCD201^+^ and FL-LSKCD201^+^ cells than to mouse PSCs (Fig. 4A). According to principal component analysis (PCA), three biological replicates of mouse PSCs, D5-LSKCD201^+^, D5-LSKCD201^−^, BM-LSKCD201^+^, and FL-LSKCD201^+^, were tightly clustered, demonstrating that the cell fractions provided reproducible transcription profiles (Fig. 4B). Alignment of reads at individual gene loci and quantification with the FPKM values confirmed that Pou5f1, Nanog, Sox2, and Esrrb expression in D5-LSKCD201^+^ and D5-LSKCD201^−^ cells was apparently silenced compared with that in mouse PSCs, and D5-LSKCD201^+^ cells had a greater reduction tendency than D5-LSKCD201^−^ cells (Fig. 4C). Global analysis also confirmed the activation of HSC transcriptional regulators, including Tal1, Erg, Gata2, Runx1, HOXB4, Hhex, Lyl1, HOXB5, Rora, Pbx1, Meis1, and Fosb, in D5-LSKCD201^+^ and D5-LSKCD201^−^ cells (Fig. 4D). Moreover, D5-LSKCD201^+^ cells expressed higher levels of the above HSC transcriptional regulators including Tal1, Erg, Gata2, HOXB4, Hhex, Lyl1, HOXB5, and Meis1 than mouse D5-LSKCD201^−^ cells (Fig. 4D). Collectively, our transcriptome analyses further support the concept that the inducible LSKCD201^+^cells from PSCs possess better hematopoiesis potential.

### Transcriptome comparison analyses of LSKCD201^+^ cells and LSKCD201^−^ cells

**Fig. 5:**
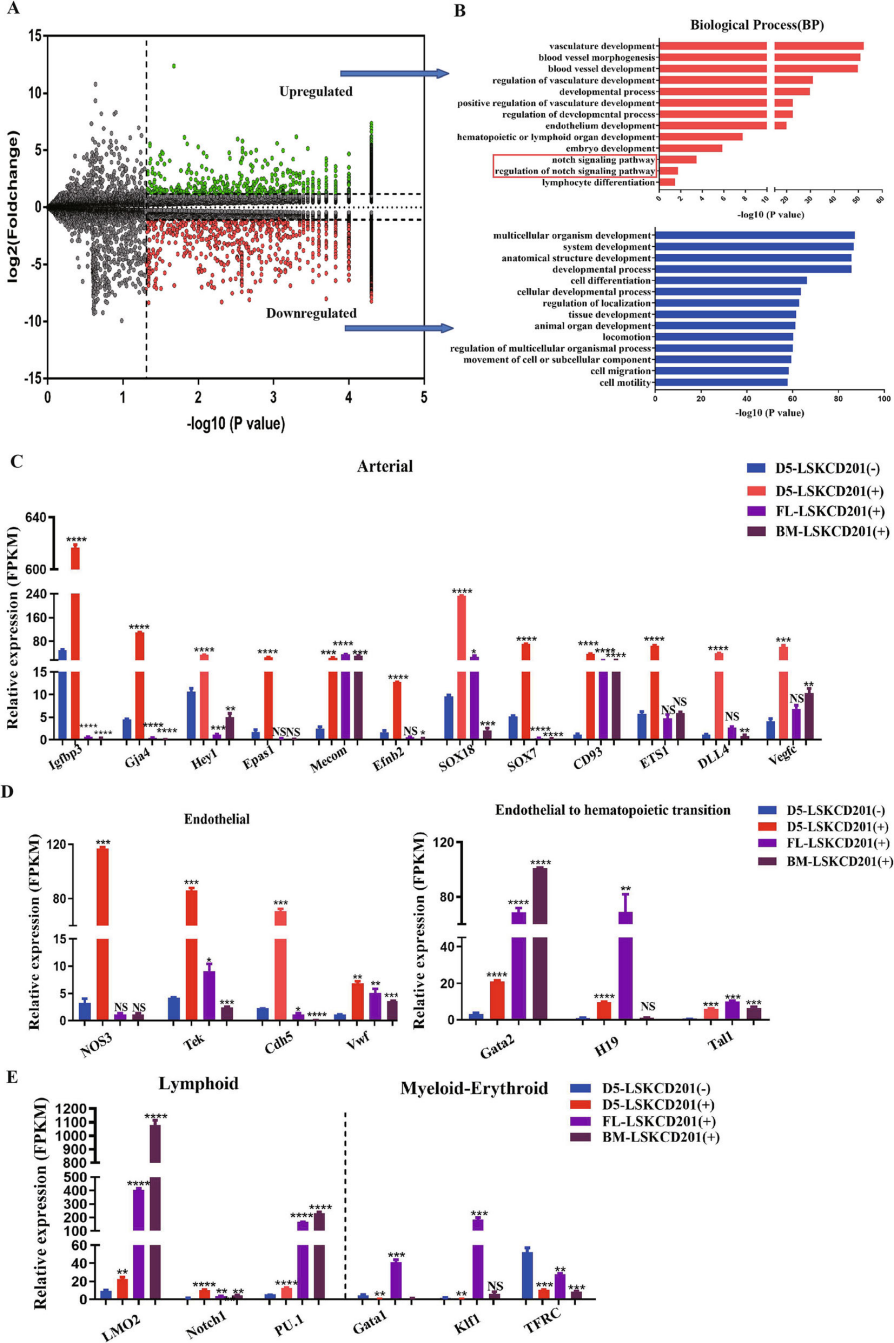
Differentiated D5-LSKCD201^+^ cells showed more hemogenic- and hematopoietic-related markers, similar to those of HSCs, than D5-LSKCD201^−^ cells. **A** Volcano plot for CD201^+^ cells and CD201^−^ cells in LSK cells on day 5 of our coculture differentiation system. The green and red dots represent 2252 and 2193 upregulated genes in the CD201^+^ and CD201^−^ cells, respectively (q< 0.05). **B** Representative statistically enriched pathways of the differentially expressed genes in CD201^+^ and CD201^−^ cells in LSK cell population. **C** The expression level of arterial-related genes determined by RNA-seq in CD201^+^ cells compared with that in CD201^−^ cells is shown as FPKM values. Data are represented as mean ± SD (*n* = 3). **D** The expression level of endothelia and EHT specific genes in CD201^+^ cells compared with that in CD201^−^ cells is shown as FPKM values. Data are represented as mean ± SD (*n* = 3). **E** The expression level of lymphoid and myeloid-erythroid biased regulator genes in CD201^+^ cells compared with that in CD201^−^ cells is shown as the FPKM values. Data are represented as mean ± SD (*n* = 3). Error bars represent mean ± SD of samples from at least three independent experiments. **p < 0.01, ***p < 0.001, ****p < 0.0001

We next aimed to further evaluate hematopoietic transcriptome differences between LSKCD201^+^ cells and LSKCD201^−^ cells. Consistent with the phenotype described above, the biological functions of genes upregulated in LSKCD201^+^ cells were more related with vasculature development (Fig. 5A, B). LSKCD201^+^ cells exhibited more apparent expression of arterial specification-associated gene SOX18, SOX7, CD93, ETS1, DLL4, Igfbp3, Gja4, Vegfc, Hey1, Epas1, Mecom, and Efnb2 [17, 18]; similar trend was presented in our study (Fig. 5C). Moreover, endothelial related genes, including NOS3, Tek, Cdh5, and Vwf had greater expression in LSKCD201^+^ cells than in LSKCD201^−^ cells (Fig. 5D). Besides, the expression levels of endothelial-to-hematopoietic transition (EHT) regulatory genes, including Gata2, Tal1, Meis1, and H19, in LSKCD201^+^ cells were higher than those in LSKCD201^-^ cells (Fig. 5D). In addition, lymphocyte potential marks the emergence of definitive hematopoietic cells, from our RNA-sequencing data analysis, we found that lymphoid-biased regulator gene LMO2, Notch1, and PU.1 obtained higher level of expression in LSKCD201^+^ cells; in contrast, myeloid-erythroid biased regulator factor Gata1, Klf1, and TFRC exhibited higher expression potential in LSKCD201^−^ cells (Fig. 5E). Together, we conclude that the LSKCD201^+^ cells derived from the self-assembling peptide-mediated coculture hematopoietic induction system exhibit stronger hematopoietic potential than the LSKCD201^−^ cells.

### LSKCD201^+^ cells from our hematopoietic differentiation system in a relatively slow cycling state

**Fig. 6:**
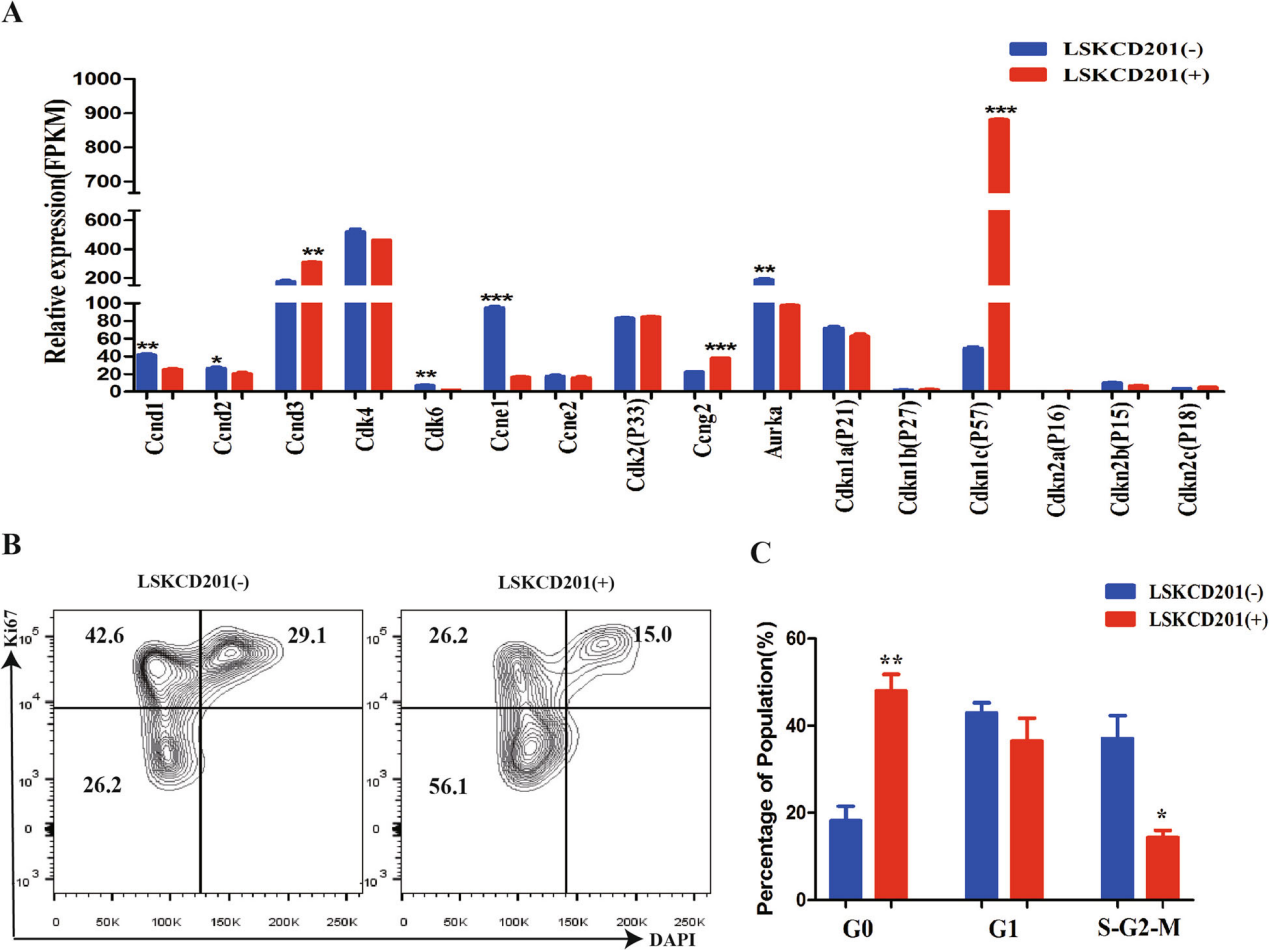
Cell cycle and mRNA expression analyses in inducible LSKCD201^+^ cells from mouse PSCs. **A** Cell cycle-related mRNA expression profiles quantitatively calculated by RNA-seq in D5-LSKCD201^+^ and D5-LSKCD201^−^ cells. **B** Representative FACS analysis of the percentage of LSKCD201^+^ cells in the Go, G1, and S-G2-M phases compared with the percentage of LSKCD201^−^ cells. **C** Statistical analysis of the percentage of LSKCD201^+^ cells in the Go, G1, and S-G2-M phases compared with the percentage of LSKCD201^−^ cells. Error bars represent mean ± SD of samples from at least three independent experiments (*n* = 3). *p < 0.05, **p < 0.01, ***p < 0.001

LSKCD201^+^ cells from fetal liver and BM have been shown to retain a relatively slow cycling state [10, 11]. Ki67, a proliferation marker, is expressed in the nucleus of cells in all phases of the activated cell cycle except for the G0 phase [19]. DAPI is a fluorescent stain that binds strongly to DNA, and its expression level reflects the amount of DNA. High-throughput RNA-seq analysis provided cell cycle-related gene profiles of LSKCD201^+^ and LSKCD201^−^ cells. Firstly, Cyclin-dependent kinase (CDK) inhibitors, such as p57, were abundantly expressed in LSKCD201^+^ cells, yet various cell cycle regulators of G1, G1/S, and G2/M phase progression, such as Ccnd1, Ccnd2, Cdk6, and Aurka were expressed at significantly higher levels in LSKCD201^−^ cells than in LSKCD201^+^ cells (Fig. 6A). We next examined the LSKCD201^+^ cell cycle status and found that compared to LSKCD201^−^ cells, LSKCD201^+^ cells had a significant increase in the G0 phase fraction and a concomitant decrease in the G1 and S/G2/M phase fractions (Fig. 6B, C). These data collectively indicate that LSKCD201^+^ cells from mouse PSCs in our hematopoietic differentiation system represent a relatively slow cycling population as compared to LSKCD201^−^ cells.

### Notch signaling is required for the generation of LSKCD201^+^ cells from mouse PSCs in our hematopoietic differentiation system

**Fig. 7:**
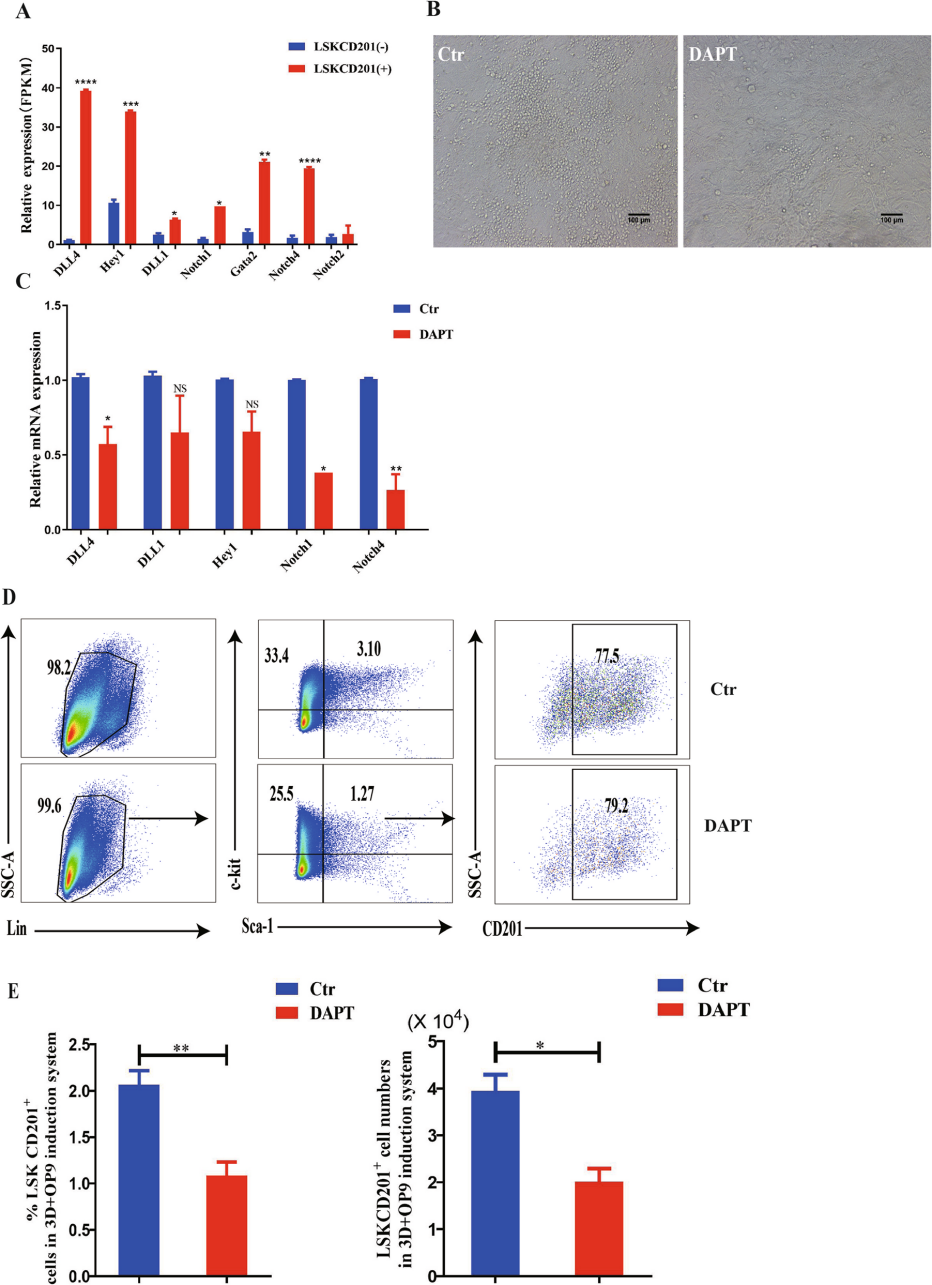
The Notch signaling pathway participates in the regulation of LSKCD201^+^ cells in our hematopoietic differentiation system. **A** The expression level of Notch signaling-related genes determined by RNA-seq in LSKCD201^+^ cells compared with the genes in LSKCD201^−^ cells (*n* = 3). **B** Representative morphology of hematopoietic cell generation on day 5 of our coculture differentiation system in the presence of DAPT or DMSO. **C** qRT-PCR analysis of Notch signaling pathway-associated genes (DLL4, DLL1, Hey1, Notch1, and Notch4) of total cells on day 5 from 3D+OP9 coculture system in the presence of DMSO (control) or DAPT. Actin used as internal control. **D** Flow cytometry analysis of the percentage of LSKCD201^+^ cells in the presence of DAPT or DMSO. **E** Statistical analysis of the percentage of LSKCD201^+^ cells in the presence of DAPT or DMSO. Error bars represent mean ± SD of samples from at least three independent experiments (*n* = 3). *p < 0.05, **p < 0.01

We next aimed to dissect the mechanism of LSKCD201^+^ cell generation from mouse PSCs. From the RNA-seq data analysis, the upregulated genes in LSKCD201^+^ cells revealed a significant association with Notch signaling pathway (Fig. 5B), and we found that the Notch signaling pathway-related genes DLL1, DLL4, Hey1, Gata2, Notch4, and Notch1 were more apparently expressed in LSKCD201^+^ cells than in LSKCD201^−^ cells (Fig. 7A). Following the above studies on the critical role of hematopoiesis regulation of the Notch signaling pathway in vivo and in vitro, we further investigated the effects of the Notch signaling pathway in the process of LSKCD201^+^ cell generation in our established differentiation system. We differentiated 3D system-derived hematopoietic-like colonies cocultured with OP9 stromal cells from day 0 to day 5 with DAPT. We observed that the emergence of hematopoietic cells in the DAPT group apparently decreased compared to that in the control group under the optical microscope (Fig. 7B). Furthermore, the expression of Notch signaling pathway-associated genes DLL4, Notch1, and Notch4 significantly decreased in the presence of DAPT as compared to DMSO (Fig. 7C). Flow cytometry data analysis confirmed that the frequencies of LSKCD201^+^ cells were reduced after Notch signaling pathway inhibition (Fig. 7D), with the statistical data showing a significant difference between the DAPT and DMSO groups (Fig. 7E). Based on these data, we concluded that the Notch signaling pathway was required for LSKCD201^+^cell generation from mouse PSCs.

## Discussion

In this study, we established a 3D self-assembling peptide-mediated hematopoietic induction system followed by coculture with OP9 stromal cells, which differentiated mouse PSCs into functional hematopoietic cells. Moreover, the new system we developed may be useful for investigating the underlying molecular mechanisms of mouse hematopoiesis. To date, the 3D system concept has been raised and applied to the field of PSC maintenance and differentiation and is now accompanied by our established 3D hematopoietic differentiation system using a self-assembling peptide biomaterial. A previous study reported that OP9 stromal cells have the ability to augment the survival of hematopoietic precursors and progenitors from human ESCs [7]. In addition, OP9 stromal cells, which can replace the complete AGM microenvironment, favor the development of definitive HSCs from pre-HSCs [8]. However, it has been difficult to generate functional HSCs that can be engrafted into adult host animals using the OP9 coculture system supplemented with only a combination of hematopoietic cytokines [20, 21]. Interestingly, in our study, using the 3D self-assembling peptide-mediated hematopoietic induction system followed by coculture with OP9 stromal cells, mouse PSCs could generate in vivo transplanted hematopoietic cells in m-NSG mouse models, reconstituting myeloid and lymphoid cells, including T and B lymphocytes, in recipient mice.

Based on a previous study, we concluded that CD201, a representative marker, can enrich mouse HSCs within the BM and fetal liver and can even enrich human HSCs within cord blood and the fetal liver [10–13]. In our established experiment, our data show that CD201 represents a novel robust marker for mouse PSC-derived hematopoietic cells. Among the LSK population derived from mouse PSCs, CD201^+^ cells had more hematopoietic cell characteristics than CD201^−^ cells. Expression of hematopoietic-regulated TFs, such as Tal1, Erg, Gata2, Hoxb4, Hhes, Lyl1, Hoxb5, Rora, Pbx1, Meis1, and FosB, was higher in the LSKCD201^+^ cells than in LSKCD201^−^ cells. The Tal1, Gata2, and Erg TFs combined with LMO2 and RUNX1c have been demonstrated to direct the reprogramming of murine fibroblasts into hematopoietic progenitor cells [22]. FosB is specifically enriched in specific HSCs [23]. Hoxb4 has been reported to confer a definitive hematopoiesis reconstitution potential on ESC-derived hematopoietic cells [24]. Pbx1 and Meis1 participate in the regulation of HSC self-renewal by maintaining HSC quiescence by forming heterodimeric and heterotrimeric complexes with HOX proteins [1]. In another study, overexpression of Erg, Rora, Hoxa9, Sox4, and Myb imparted transient myeloerythroid engraftment potential onto induced pluripotent stem cell (iPSC)-derived blood cell progenitors [25]. Hoxb5 has been reported to mark long-term mouse HSCs within the BM [26]. Gata2, as a target of Lyl1, has been implicated in the regulation of HSC survival and proliferation [27]; in addition, Gata2 plays a critical role in regulating endothelial-to-hematopoietic transition from PSCs hematopoietic differentiation [28]. Based on transcriptome analyses by our RNA-seq data, the aforementioned hematopoietic-regulated TF expression levels within LSKCD201^+^ cells from our hematopoietic induction system displayed similarity with LT-HSC within mouse BM and fetal liver, yet the question involving how the TFs together regulate the function of LSKCD201^+^ cells would be resolved in the future research.

Regarding the hematopoietic potential of LSKCD201^+^ cells derived from our differentiation system in vitro, CD201^+^ cell populations possess stronger hematopoietic predominance than CD201^−^ cell populations. First, LSKCD201^+^ cell populations obtained higher expression of endothelial and hematopoietic-related markers, such as Pecam1, Cdh5, Itga2b, Cd34, and CD47, than LSKCD201^−^ cell populations (Figure S2A). In addition, EHT-related regulatory factors, such as Gata2, Tal1, and H19, were more apparently expressed in LSKCD201^+^ cell populations than in LSKCD201^−^ cell populations. However, the individual role of each EHT factor in the generation of LSKCD201^+^ cells needs to be investigated in detail in future work. Hox genes have been reported as regulators of HSC self-renewal and differentiation [29]. In our study, we found that Hoxb cluster (Hoxb2, Hoxb4, Hoxb5, Hoxb7) and Hoxa cluster (Hoxa13 and Hoxa11os) gene expression was much higher in CD201^+^ cell populations than in CD201^−^ cell populations (Figure S2B). However, Hoxa9, a critical TF that promotes the hematopoietic commitment of human ESCs, has lower expression in mouse PSCs-derived LSKCD201^+^ cell populations than in LSKCD201^-^ cell populations (data not shown). The relative molecular mechanism of Hoxb cluster and Hoxa cluster genes for CD201^+^ cell populations derived from PSCs remains elusive. From in vitro long-term culture observations, such as colony-forming unit assays, LSKCD201^+^ cells obtained from our induction system have a higher proliferative potential than LSKCD201^−^ cells, maybe attributing to the higher expression of Hox genes and EHT genes, which need our further research in the future.

In our study, our findings showed that using a 3D self-assembling peptide-mediated hematopoietic induction system followed by coculture with OP9 stromal cells could produce many more LSKCD201^+^ cells from mouse PSCs than the control system followed by coculture with 0.1% gelatin. Our data demonstrated that these LSKCD201^+^ cells were more likely to be in a slow cycling state than LSKCD201^−^ cells under the regulation of CDK inhibitors, such as p57. Whereas various regulators of G1, G1/S, and G2/M cell cycle phase progression, Ccnd1, Ccnd2, Cdk6, and Aurka, were expressed at significantly higher levels in LSKCD201^−^ cells than in LSKCD201^+^ cells. These characteristics were confirmed in CD201^+^ cells isolated from mouse fetal liver and adult BM. The OP9 stromal cell-mediated ECM-rich microenvironment may serve as a niche for LSKCD201^+^ cells that are in a relatively slow cycling state. Our RNA-seq data showed that genes upregulated in 3D+OP9 coculture system were enriched in extracellular matrix organization signaling pathway. However, the detailed regulatory mechanism will be addressed in future studies.

Currently, the in vivo engraftment potential of mouse PSC-derived hematopoietic cells in vitro still faces great challenges, which are attributed to the difficulty of generating a high yield of truly functional induced HSCs similar to mouse embryonic or adult HSCs [4]. Recent studies have reported c-kit expression as a potential marker of the repopulating activity of induced hematopoietic cells [30]. To date, it is the only cell-surface marker that has been shown to be expressed on all HSCs throughout embryonic development and adulthood [30]. In our induction system, we obtained high-efficiency c-kit^+^ hematopoietic cells. Most importantly, our transplantation data analysis confirmed that PSC-derived hematopoietic cells exhibited a hematopoiesis reconstitution potential for up to 4 weeks in both the PB and BM of sublethally irradiated (2.0 Gy) m-NSG mice injected intrafemorally with c-kit^+^ cells. Because the mice were in such a poor state, only a limited number survived. Here, we only could provide representative data that showed donor-derived cells in peripheral blood of m-NSG in 3D+OP9 induction system after 6 weeks’ transplantation (Figure S3). Unfortunately, we failed to detect in vivo hematopoiesis in lethally irradiated C57BL/6 (CD45.1) mice injected intrafemorally with these cell populations, and the mice died after approximately 2 weeks, which suggested that functional deficiency occurred in induced c-kit^+^ cells rather than in mouse developmental and adulthood-derived c-kit^+^ cells. In addition, the chimerism efficiency detected in m-NSG mice transplanted with c-kit^+^ cells from the 3D+OP9 coculture protocol was higher compared with that in mice transplanted with cells from 3D+0.1% gelatin coculture, maybe reasoned by the fact that the transplanted cells derived from the group of 3D+OP9 coculture composed of larger quantity of CD201^+^c-kit^+^ hematopoietic cells, which require our future study. Whatever, hematopoietic cells from the 3D self-assembling peptide-mediated OP9 or 0.1% gelatin coculture system possess hematopoiesis reconstitution superiority in vivo, which provide a desirable in vitro hematopoietic development platform via mouse PSCs model. Further work will focus on improving and optimizing the culture conditions for the maintenance and expansion of these functional repopulating cells, which may not only provide a useful tool to study HSC development but also lay the foundation for the clinical application of PSCs-derived hematopoietic cells.

## Conclusion

In our study, we established an in vitro differentiation system yielding in vivo hematopoiesis hematopoietic cells from mouse PSCs through a 3D induction system followed by coculture with OP9 stromal cells. In addition, we identified the high percentage of CD201^+^ cluster owning the more hematopoiesis superiority in terms of hematopoietic transcriptome level, CFU assays, and cell cycle. Meanwhile, we demonstrated the critical regulatory role of Notch signaling pathway necessary for in vitro CD201^+^ hematopoietic cell generation from mouse PSCs.

Together, our findings advance the understanding of mouse hematopoietic development; in addition, this study contributed to the mechanistic understanding of mouse PSC hematopoietic differentiation.

## Supplementary Information

**Additional file 1: Figure S1** shows the CD201 expression in our 3D self-assembling peptide-mediated OP9 co-culture hematopoietic induction system. (A) Flow cytometry analysis of the percentage of CD201^+^ on day2, day5 and day7 respectively. (B) Statistical analysis of the percentage of CD201^+^ in 3D+OP9 hematopoietic induction system. Data are represented as mean ± SD (*n* = 3).

**Additional file 2: Figure S2** is the hematopoietic related gene expression in between CD201^+^ cells and CD201^-^ cells. (A) The expression level of hematopoietic markers in CD201^+^ cells compared with that in CD201^-^ cells is shown as FPKM values, Data are represented as mean ± SD (*n* = 3). (B) The expression level of HOX family genes in CD201^+^ cells compared with that in CD201^-^ cells is shown as FPKM values, Data are represented as mean ± SD (*n* = 3).

**Additional file 3: Figure S3** demonstrates representative flow cytometric plots for CD45.1 and CD45.2 expression in the PB from m-NSG recipient mice (CD45.1) after 6 weeks transplantation, meanwhile representative flow cytometric plots for expression of CD11b, CD19 and thy1.2 in gated CD45.2^+^ cells.

## Data Availability

The data that support the findings of this study can be obtained from the corresponding author upon reasonable request.

## Abbreviations

PSCs: Pluripotent stem cells (PSCs)
HSCs: Hematopoietic stem cells
HSPCs: Hematopoietic stem and progenitor cells
TF: Transcription factor
EBs: Embryonic bodies
iHSC: Inducible HSC
LTR: Long-term reconstitution
ESCs: Embryonic stem cells
AGM: Aorta-gonad-mesonephros
iHCs: Induced hematopoietic cells
PB: Peripheral blood
BM: Bone marrow
EPCR: Endothelial protein C receptor
CFU: Colony forming unit
MEFs: Mouse embryonic fibroblasts
DAPI: 4′,6-Diamidino-2-phenylindole
LSK: Lineage-negative Sca-1^+^c-kit^+^
CFU-G: CFU-granulocyte
CFU-M: CFU macrophage
CFU-GM: CFU granulocyte macrophage
CFU-GEMM: CFU granulocyte erythrocyte monocyte macrophage
DAPT: Dual antiplatelet therapy
iPSC: Induced pluripotent stem cell
E12.5: Embryonic day 12.5
m-NSG: NOD-Prkdc^scid^IL2rg^null^
