# Supplementary figures and images for: Enhanced HSC-like cell generation from mouse pluripotent stem cells in a 3D induction system cocultured with stromal cells
Source: Stem Cell Res Ther. 2021 Jun 19;12:353. doi: 10.1186/s13287-021-02434-2 (PMC8214308; doi:10.1186/s13287-021-02434-2)

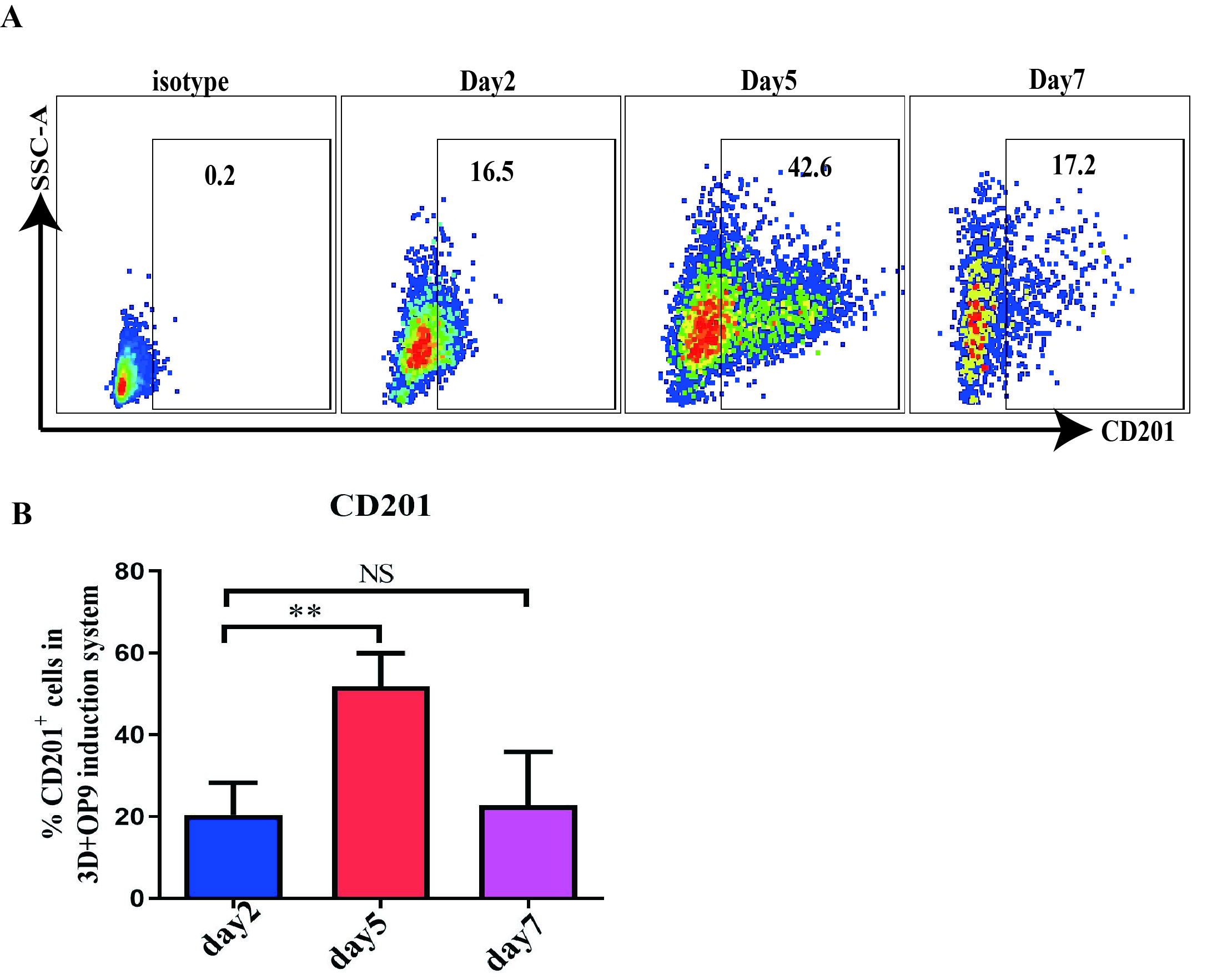

Supplement: Supplementary file 1 — Additional file 1: Figure S1 shows the CD201 expression in our 3D self-assembling peptide-mediated OP9 co-culture hematopoietic induction system. (A) Flow cytometry analysis of the percentage of CD201+ on day2, day5 and day7 respectively. (B) Statistical analysis of the percentage of CD201+ in 3D+OP9 hematopoietic induction system. Data are represented as mean ± SD (n = 3). [file 13287_2021_2434_MOESM1_ESM.jpg]

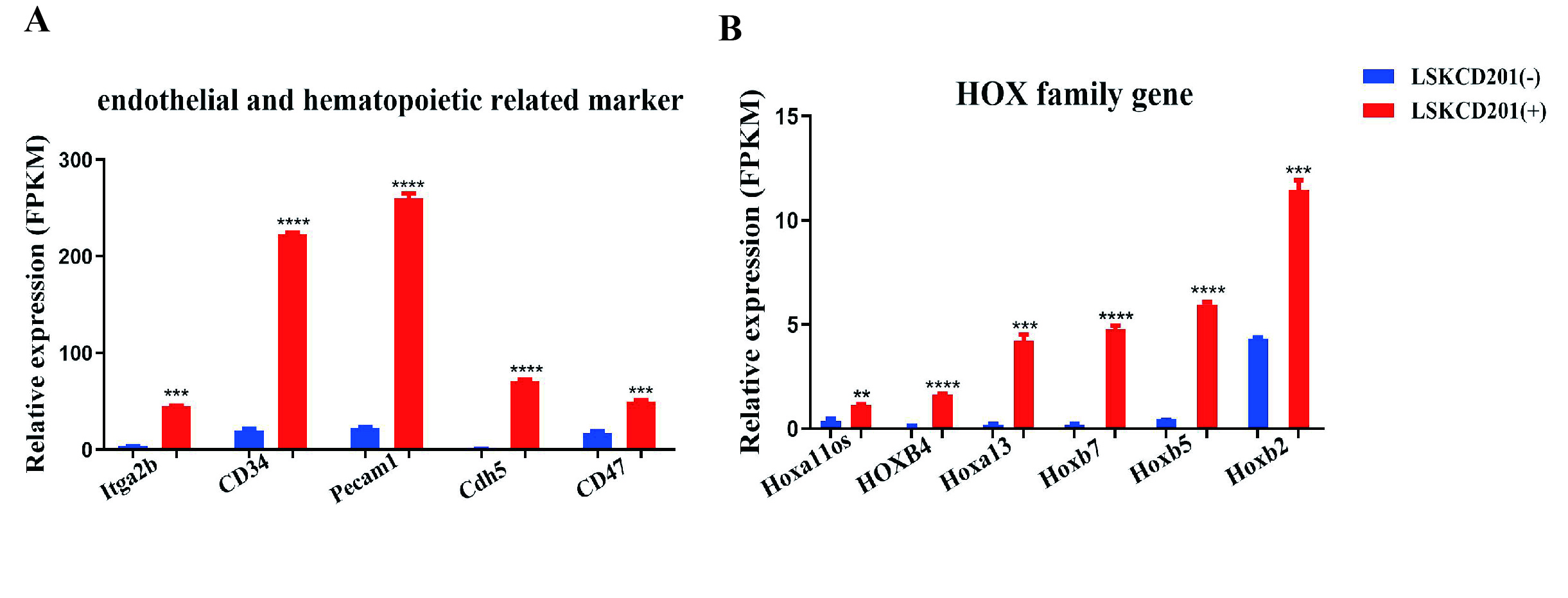

Supplement: Supplementary file 2 — Additional file 2: Figure S2 is the hematopoietic related gene expression in between CD201+ cells and CD201- cells. (A) The expression level of hematopoietic markers in CD201+ cells compared with that in CD201- cells is shown as FPKM values, Data are represented as mean ± SD (n = 3). (B) The expression level of HOX family genes in CD201+ cells compared with that in CD201- cells is shown as FPKM values, Data are represented as mean ± SD (n = 3). [file 13287_2021_2434_MOESM2_ESM.jpg]

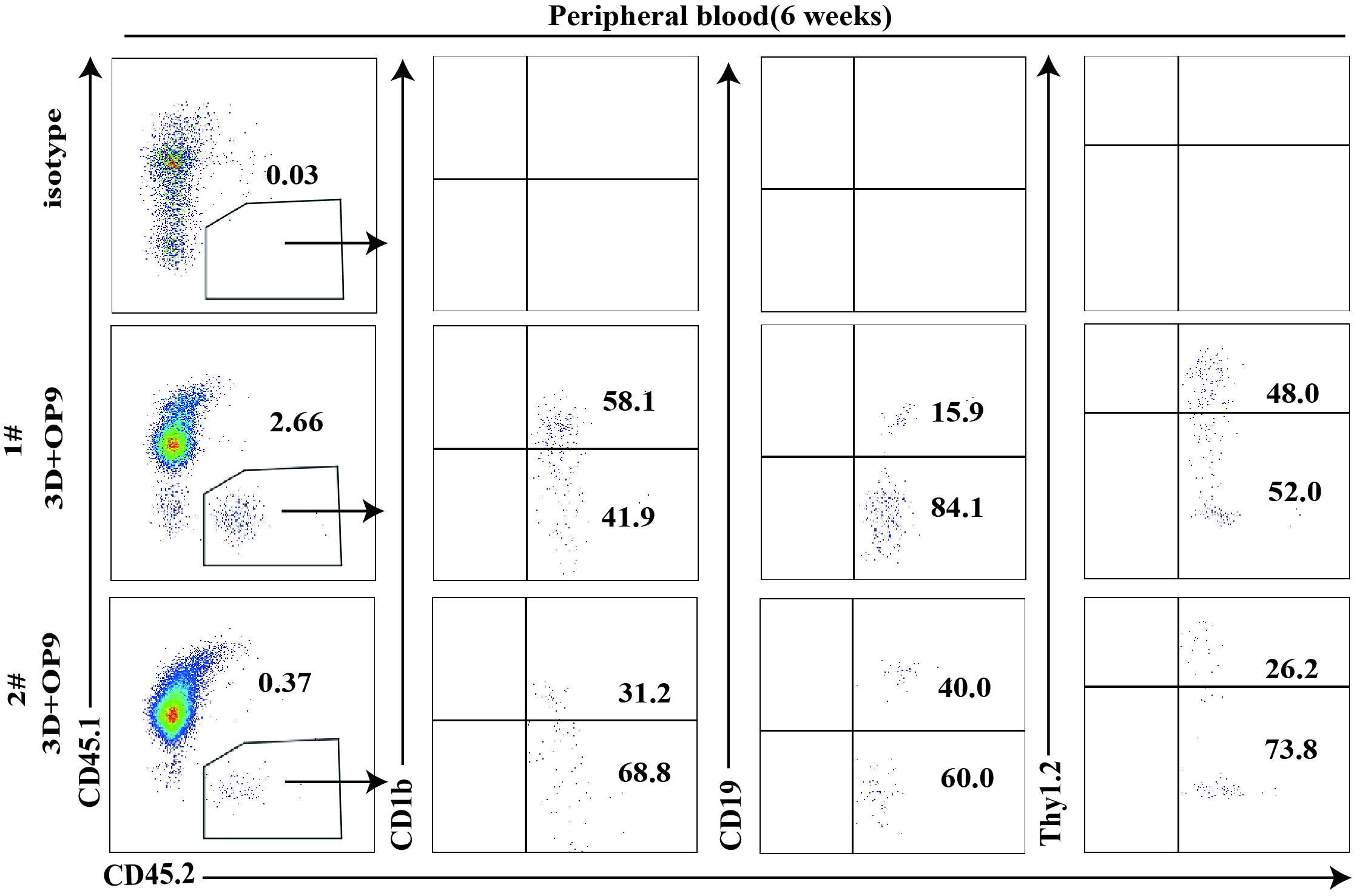

Supplement: Supplementary file 3 — Additional file 3: Figure S3 demonstrates representative flow cytometric plots for CD45.1 and CD45.2 expression in the PB from m-NSG recipient mice (CD45.1) after 6 weeks transplantation, meanwhile representative flow cytometric plots for expression of CD11b, CD19 and thy1.2 in gated CD45.2+ cells. [file 13287_2021_2434_MOESM3_ESM.jpg]
